# Surgical site infections following colorectal cancer surgery: a randomized prospective trial comparing common and advanced antimicrobial dressing containing ionic silver

**DOI:** 10.1186/1477-7819-10-94

**Published:** 2012-05-23

**Authors:** Roberto Biffi, Luca Fattori, Emilio Bertani, Davide Radice, Nicole Rotmensz, Pasquale Misitano, Sabine Cenciarelli, Antonio Chiappa, Liliana Tadini, Marina Mancini, Giovanni Pesenti, Bruno Andreoni, Angelo Nespoli

**Affiliations:** 1 Division of Abdomino-Pelvic and Minimally Invasive Surgery, European Institute of Oncology, Via G. Ripamonti, Milan, 435-20141, Italy; 2 Division of General Surgery, San Gerardo Hospital, University of Milano-Bicocca, Monza, Italy; 3 Division of General and Laparoscopic Surgery, European Institute of Oncology, Milan, Italy; 4 Division of Epidemiology and Biostatistics, European Institute of Oncology, Milan, Italy; 5 Department of Patient Care, European Institute of Oncology, Milan, Italy

**Keywords:** Colorectal cancer, Elective surgery, Hydrofiber dressing, Ionic silver, Surgical site infection

## Abstract

**Background:**

An antimicrobial dressing containing ionic silver was found effective in reducing surgical-site infection in a preliminary study of colorectal cancer elective surgery. We decided to test this finding in a randomized, double-blind trial.

**Methods:**

Adults undergoing elective colorectal cancer surgery at two university-affiliated hospitals were randomly assigned to have the surgical incision dressed with Aquacel® Ag Hydrofiber dressing or a common dressing. To blind the patient and the nursing and medical staff to the nature of the dressing used, scrub nurses covered Aquacel® Ag Hydrofiber with a common wound dressing in the experimental arm, whereas a double common dressing was applied to patients of control group. The primary end-point of the study was the occurrence of any surgical-site infection within 30 days of surgery.

**Results:**

A total of 112 patients (58 in the experimental arm and 54 in the control group) qualified for primary end-point analysis. The characteristics of the patient population and their surgical procedures were similar. The overall rate of surgical-site infection was lower in the experimental group (11.1% center 1, 17.5% center 2; overall 15.5%) than in controls (14.3% center 1, 24.2% center 2, overall 20.4%), but the observed difference was not statistically significant (*P* = 0.451), even with respect to surgical-site infection grade 1 (superficial) versus grades 2 and 3, or grade 1 and 2 versus grade 3.

**Conclusions:**

This randomized trial did not confirm a statistically significant superiority of Aquacel® Ag Hydrofiber dressing in reducing surgical-site infection after elective colorectal cancer surgery.

**Trial registration:**

Clinicaltrials.gov: NCT00981110

## Background

Surgical-site infection (SSI) occurs in 300,000 to 500,000 patients who undergo surgery in the US each year. For surgical patients, SSI are the most common hospital-acquired infection. In clean-contaminated and contaminated surgery, such as elective major colorectal surgery, there has been wide discrepancy in the reported incidence of incisional SSI, ranging from 3% to 30% [1,2], reflecting differences in diagnostic criteria and follow-up applied. SSI is associated with significant attributable morbidity and mortality, prolonged hospital length of stay, and a high cost to the patient and the institution. Several studies have suggested a doubling of the mortality rate, with an annual cost of $1.8 billion to the US health care system and £1 billion to the National Health Service in England [3,4]. Because the patient’s skin is a major source of pathogens, it is conceivable that improving skin antisepsis would decrease SSIs; a large randomized trial has shown that preoperative cleansing of the patient’s skin with chlorhexidine-alcohol is superior to cleansing with povidone-iodine for preventing surgical-site infection after clean-contaminated surgery, including colorectal procedures [5].

Ionic silver (Ag+), which is the oxidized active state of silver, has received renewed interest and research for use as a prophylactic antimicrobial agent in wound dressings due to its broad spectrum antibacterial range, including aerobic, anaerobic, Gram-negative and Gram-positive bacteria, as well as yeast and fungi [6-8]. Aquacel® Ag Hydrofiber dressing (ConvaTec, Princeton, NJ, USA) is a moisture retention dressing that consists of soft non-woven sodium carboxymethylcellulose fibers combined with 1.2% ionic silver, which is distributed throughout the dressing material. The concept of Aquacel® Ag Hydrofiber dressing is to retain the hydrofiber’s physical properties with the additional benefits of ionic silver, which is slowly released into the wound, creating a moist antimicrobial environment. Multiple clinical studies have been performed to assess its effectiveness for the treatment of a variety of wounds, managed in acute and chronic settings. Nevertheless, a recent Cochrane systematic review failed to find an advantage for silver-containing wound dressings after analyzing 26 randomized controlled trials that compared silver-containing wound dressings and topical agents with silver-containing and non-silver-containing comparators on uninfected wounds. The authors of the review concluded that there is insufficient evidence to establish whether silver-containing dressings or topical agents promote wound healing or prevent wound infection [9]. No published randomized studies have previously examined the effect of silver-containing wound dressing on the incidence of SSI. The average baseline rate of SSI after colorectal cancer elective surgery at the European Institute of Oncology was 23% with common postoperative dressing; a pilot prospective non-randomized study of 100 patients showed a reduction of this rate to 4% by substituting the Aquacel® Ag Hydrofiber dressing for the common dressing [10]. We decided to test this preliminary finding in a prospective, randomized, double-blind trial against a common dressing as control, having the main objective to compare the efficacy of Aquacel® Ag Hydrofiber dressing with that of a common postoperative one for preventing SSIs in colorectal cancer elective surgery.

## Methods

### Study design

We conducted this prospective, randomized trial between June 2008 and September 2010 at two university-affiliated hospitals in Italy (IEO, European Institute of Oncology, Milano and University of Milano-Bicocca at San Gerardo Hospital, Monza). The institutional review board and Ethics Committee at each hospital approved the study protocol, and written informed consent was obtained from all patients before enrollment. The trial was initially registered at Central Registry of Randomized Clinical Trials of the Italian Health Ministry, and then received registration no. NCT00981110 by clinicaltrials.gov.

### Patients

Patients aged 18 to 75 years who were undergoing colorectal cancer elective surgery by laparotomic approach were eligible for enrollment. Exclusion criteria were a history of allergy to dressing components, evidence of active infection at or adjacent to the operative site, coagulopathy (defined as platelet count less than 50,000 cells/μL or a prothrombin time more than 18 seconds), intestinal obstruction, active bowel bleeding, life expectancy less than 6 months, inability to give written informed consent or a program of minimally invasive surgery (laparoscopy or robot- assisted).

### Interventions

Enrolled patients were randomly assigned to have the surgical incision dressed with Aquacel Ag Hydrofiber dressing (ConvaTec) in the experimental arm, or a common dressing (Mepore, Molnlycke Health Care, Gothenburg, Sweden) in the control arm. To help match the two groups and address potential inter-hospital differences, randomization was stratified by hospital with the use of computer-generated randomization numbers without blocking. In order to maintain the double-blind characteristic of this trial, some actions were taken. First, the generator of the assignment was a data manager, who was separated from the executor; second, dressings were applied by the scrub nurses in the operating theatre at the end of each procedure. The Aquacel Ag Hydrofiber dressing was covered by a common wound dressing in the experimental arm, whereas a double common dressing was applied to patients of the control group to blind the patient, the nursing and the medical staff and the independent data collector as to the nature of the dressing used. All patients received a preoperative scrub and then painting with an aqueous solution of 10% povidone-iodine; all patients had mechanical bowel preparation and antibiotic prophylaxis in agreement with predefined protocols.

### Efficacy outcomes

The primary end-point of the study was the occurrence of any SSI within the 30 days after surgery. The operating surgeon became aware of which dressing had been applied only after the superficial dressing was removed on the seventh postoperative day. Similarly, both the patients and the investigators who diagnosed SSI on the basis of criteria developed by the Centers for Disease Control and Prevention (CDC) remained unaware of the group assignments until removal of the dressing. Secondary end-points included the occurrence of individual types of SSIs. These were classified as superficial incisional infection (which involved only the skin and subcutaneous tissue and excluded stitch-related abscesses), deep incisional infection (which involved fascia and muscle), or organ-space infection (which involved any organ or space other than the incised layer of body wall that was opened or manipulated during the operation).

### Clinical assessment

The surgical site and the patient’s vital signs were assessed at least once a day during hospitalization, on discharge and at the time of follow-up evaluation (30th postoperative day). Dressings were removed on seventh postoperative day as per protocol, or earlier whenever SSI was suspected. Data from the assessment and follow-up of these patients were entered into a software registry and analyzed by an epidemiologist-biostatistician (DR). After discharge, the investigators called the patients once a week during the 30-day follow-up period and arranged for prompt clinical evaluation if infection was suspected. Whenever SSI was suspected or diagnosed, clinically relevant microbiologic samples were cultured. Investigators, who were unaware of the patients’ group assignments, assessed the seriousness of all adverse events and determined whether they were related to the study.

### Statistical analysis

The average baseline rate of SSI at the participating hospitals was 23% after colorectal cancer elective surgery with standard postoperative dressing, and we estimated that substituting the Aquacel Ag Hydrofiber dressing for common would reduce this rate to 4%. This was the overall SSI rate we detected in a pilot prospective non-randomized study of 100 patients with colorectal cancer who had elective surgery at the European Institute of Oncology in 2007 [10]. Therefore, we planned to enroll 56 patients in each study group for the study to have 80% power to detect a 19% difference in the rates of SSI between the two groups, using a two-sided Fisher’s exact test at a two-tailed significance level of *P ≤*0.05. The significance of differences between the two study groups in terms of patient characteristics was determined with the use of the Wilcoxon rank-sum test for continuous variables in case of non-normally distributed data, unpaired *t*-test otherwise. The center-effect on continuous variables was tested using an analysis of variance (ANOVA) model with center and treatment arm as the main effects without interaction terms. Between-group comparisons for categorical variables were done using either the Fisher’s exact test or the Cochran-Mantel-Haenszel Chi-square test stratified by center as appropriate. Furthermore, between-group comparisons for efficacy outcomes (for example, the risk of any SSI) were done by estimating the univariate (unadjusted) and multivariate (adjusted) odds-ratios in a logistic regression analysis. Odds-ratios are tabulated alongside their 95% confidence interval (CI).

The assessed risk factors were prespecified in the protocol, and the statistical methods were preplanned. All analyses were conducted using SAS 9.2 software (Cary, NC, USA).

## Results

### Patients

**Figure 1:**
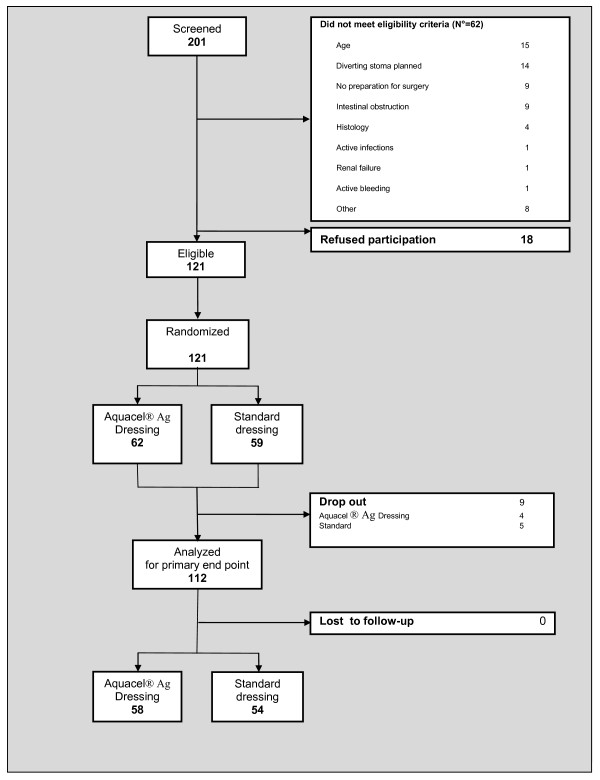
Trial profile, conforming to the Consolidated Standards of Reporting Trials guidelines.

A total population of 201 patients were screened for eligibility during the 16-month accrual period of the study, and 121 were randomly assigned to a study group: 62 to the Aquacel Ag Hydrofiber dressing group and 59 to the control dressing group. Nine patients dropped out of the study after surgery (four in the Aquacel Ag Hydrofiber and five in the control dressing group). Therefore, 112 patients (58 in the Aquacel Ag Hydrofiber dressing group and 54 in the control dressing group) were included in the analyses. A trial profile, conforming to the Consolidated Standards of Reporting Trials guidelines, is shown in Figure 1.

The patients in the two study groups were similar with respect to their demographic characteristics, type of tumor (colon versus rectal), body mass index (BMI), American Society of Anesthesiologists (ASA) score and risk factors for infection (smoking status, preoperative chemoradiation therapy). None of the considered variables differed with respect to surgical center; only surgical wound length differed, as the San Gerardo Monza Center showed a significantly (*P* <0.0001 ANOVA F-test) longer length (Tables 1 and 2). All patients received systemic prophylactic antibiotics within the 30 minutes before the initial incision, and there were no significant differences in the type or number of antibiotics given to the two study groups (data not shown).

**Table 1:** Patients’ characteristics

**Characteristic**	**Arm**	**N**	**Mean ± SD**	**Median**	**Minimum and maximum**	***P***^**a**^
**Age (years)**	A	54	62.9 ± 9.0	64.0	35,76	0.593
	B	58	63.6 ± 9.2	63.5	38,77	
	*Overall*	*112*	*63.3 ± 9.0*	*64.0*	*35,77*	
**Body mass index (kg/m**^**2**^**)**	A	54	25.4 ± 4.4	25.1	18,36	0.747
	B	58	25.7 ± 4.3	24.9	19,39	
	*Overall*	*112*	*25.5 ± 4.3*	*25.0*	*18,39*	
**Wound length (cm)**	A	46	22.3 ± 7.2	20.0	14,44	0.154
	B	48	19.9 ± 6.3	20.0	10,40	
	*Overall*	*94*	*21.0 ± 6.8*	*20.0*	*10,44*	

^a^Wilcoxon or unpaired *t*-test as appropriate. A: standard dressing; B: Aquacel Ag Hydrofiber dressing; SD: standard deviation.

**Table 2:** Patients’ characteristics

**Characteristic**		**A**^**a**^	**B**^**b**^	**Overall**	***P***^**a**^
		**N (%)**	**N (%)**		
**Gender**	Female	25 (46.3)	22 (37.9)	47 (42.0)	0.444
	Male	29 (53.7)	36 (62.1)	65 (58.0)	
**Diagnosis**	Colon	36 (66.7)	41 (70.7)	77 (68.8)	0.687
	Rectal	18 (33.3)	17 (29.3)	17 (15.2)	
**Preoperative treatment**	CT+RT	7 (13.0)	7 (12.1)	14 (12.5)	1.000
	None	47 (87.0)	51 (87.9)	98 (87.5)	
**Smoking status**	Never	29 (53.7)	28 (48.3)	57 (50.9)	
	Previous	18 (33.3)	20 (34.5)	38 (33.9)	0.759
	Smoker	7 (13.0)	10 (17.2)	17 (15.2)	
**ASA**	I	4 (7.4)	3 (5.2)	7 (6.3)	
	II	40 (74.1)	46 (79.3)	86 (76.8)	0.787
	III	10 (18.5)	9 (15.5)	19 (17.0)	

^a^Chi-square of Fisher’s exact test as appropriate; A: standard dressing; ASA: American Society of Anesthesiologists score; B: Aquacel Ag Hydrofiber dressing; CT+RT: preoperative chemoradiation therapy.

### Rates of infection

Table 3 summarizes the main results. The overall rate of SSI was lower in the experimental group, but the observed difference was not statistically significant: there were nine (15.5%) SSIs of any grade in the experimental group and 11 (20.4%) in controls (*P* = 0.623). Infections rates did not significantly differ with respect to surgical center (experimental arm: 11.1% center 1, 17.5% center 2, overall 15.5%; controls: 14.3% center 1, 24.2% center 2, overall 20.4% (data not shown), Cochran-Mantel-Haenszel Stratified Test: *P* = 0.451).

**Table 3:** Primary outcome - surgical-site infections

**Surgical-site infection grade**	**A**	**B**	**Overall**	***P***^**a**^
	**N (%)**	**N (%)**		
**None**	43 (79.6)	49 (84.5)	92 (82.1)	
**1**	8 (14.8)	5 (8.6)	13 (11.6)	0.802
**2**	2 (3.7)	3 (5.2)	5 (4.5)	
**3**	1 (1.9)	1 (1.7)	2 (1.8)	
**None**	43 (79.6)	49 (84.5)	92 (82.1)	0.623
**Any**	11 (20.4)	9 (15.5)	20 (17.9)	
**None**	43 (79.6)	49 (84.5)	92 (82.1)	
**1**	8 (14.8)	5 (8.6)	13 (11.6)	0.653
**2,3**	3 (5.6)	4 (6.9)	7 (6.3)	
**None**	43 (79.6)	49 (84.5)	92 (82.1)	
**1,2**	10 (18.5)	8 (13.8)	18 (16.1)	0.803
**3**	1 (1.9)	1 (1.7)	2 (1.8)	

^a^Chi-square or Fisher’s exact test as appropriate. A: standard dressing; B: Aquacel Ag Hydrofiber dressing.

There were no differences even with respect to SSI grade 1 versus 2 and 3 or grade 1 and 2 versus 3 (Table 3). Use of Aquacel Ag Hydrofiber dressing was not associated with significantly fewer superficial incisional infections or deep infections. As expected, there were no significant differences between the two study groups in the incidence of organ-space infection (SSI grade 3). Table 4 shows some characteristics of the clinical assessment of the surgical wound, including presence of necrosis, erythema, edema and hematoma; none of these findings differed between groups, along with detection of fever, shiver or leukocytosis. Similarly, the need of an anticipated change of the dressing was not significantly more frequent in the control arm.

**Table 4:** Other outcomes

**Characteristic**		**A**	**B**	**Overall**	***P***^**a**^
		**N (%)**	**N (%)**		
**Necrosis**	Fair	0	2 (3.5)	2 (1.8)	
	Light	1 (1.9)	0	1 (0.9)	0.361
	None	53 (98.1)	56 (96.5)	109 (97.3)	
**Erythema**	Fair	2 (3.7)	0	2 (1.8)	
	Light	4 (7.4)	9 (15.5)	13 (11.6)	0.158
	None	48 (88.9)	49 (84.5)	97 (86.6)	
**Edema**	Light	5 (9.3)	3 (5.2)	8 (7.1)	0.479
	None	49 (90.7)	55 (94.8)	104 (92.9)	
**Hematoma**	No	51 (94.4)	53 (91.4)	104 (92.9)	0.718
	Yes	3 (5.6)	5 (8.6)	8 (7.1)	
**Fever**	No	47 (87.0)	47 (81.0)	94 (83.9)	0.447
	Yes	7 (13.0)	11 (19.0)	18 (16.1)	
**Shiver**	No	51 (94.4)	55 (94.8)	106 (94.6)	1.000
	Yes	3 (5.6)	3 (5.2)	6 (5.4)	
**Leukocytosis**	No	45 (83.3)	48 (82.8)	93 (83.0)	1.000
	Yes	9 (16.7)	10 (17.2)	19 (17.0)	
**Infection within 30 days**	No	46 (85.2)	52 (89.7)	98 (87.5)	0.572
	Yes	8 (14.8)	6 (10.3)	14 (12.5)	

^a^Chi-square of Fisher’s exact test as appropriate. A: standard dressing; ASA: American Society of Anesthesiologists score; B: Aquacel Ag Hydrofiber dressing.

Table 5 reports univariate risk estimates of acquiring a SSI in the study groups; only surgery for rectal cancer was possibly significantly related to an increased risk of SSI (unadjusted odds ratio: 2.68; 95% CI: 1.00 to 7.21; *P* = 0.051).This finding was also confirmed in the multivariate analysis (Table 6): adjusted odds ratio: 4.07; 95% CI: 1.01 to 16.4; *P* = 0.048). Study arm, gender, surgical center, smoking status, preoperative chemoradiation therapy, ASA score, age, BMI and surgical wound length did not show any impact on SSI rate.

**Table 5:** Univariate risk estimates of acquiring a surgical-site infection

**Risk factor**		**Unadjusted odds ratio**	**95% confidence interval**	***P***
**Arm**	A	Reference	-	
	B	0.72	0.27,1.90	0.504
**Gender**	Male	Reference	-	
	Female	0.40	0.13,1.18	0.097
**Center**	European Institute of Oncology	Reference	-	
	San Gerardo Monza	0.57	0.19,1.70	0.313
**Smoking status**	Never	Reference	-	
	Previous	1.42	0.50,4.09	0.598
	Smoker	1.14	0.27,4.80	0.951
**Diagnosis**	Colon	Reference		
	Rectal	2.68	1.00,7.21	***0.051***
**Preoperative treatment**	None	Reference	-	
	CT+RT	2.05	0.57,7.35	0.271
**ASA**	I	Reference	-	
	II	1.27	0.14,11.3	0.998
	III	1.60	0.15,17.4	0.658
**Age**^**a**^		1.02	0.96,1.08	0.564
**Body mass index**^**a**^		1.08	0.97,1.21	0.155
**Wound length**^**a**^		0.96	0.88,1.05	0.389

^a^Odds-ratio estimated by unit increase. A: standard dressing; B: Aquacel Ag Hydrofiber dressing; CT+RT: preoperative chemoradiation therapy.

**Table 6:** Multivariate risk estimates of acquiring a surgical-site infection

**Risk factor**		**Adjusted odds ratio**	**95% confidence interval**	***P***
**Arm**	A	Reference	-	
	B	0.68	0.20,2.31	0.539
**Gender**	Male	Reference	-	
	Female	0.86	0.22,3.36	0.828
**Center**	European Institute of Oncology	Reference	-	
	San Gerardo Monza	0.89	0.17,4.70	0.893
**Smoking status**	Never	Reference	-	
	Previous	0.54	0.13,2.26	0.332
	Active	1.35	0.19,9.74	0.539
**Diagnosis**	Colon	Reference		
	Rectal	4.07	1.01,16.4	***0.048***
**Preoperative treatment**	None	Reference	-	
	CT+RT	1.96	0.26,14.9	0.516
**ASA**	I	Reference	-	
	II	0.25	0.01,4.59	0.224
	III	0.57	0.03,11.4	0.903
**Age**^**a**^		1.05	0.97,1.14	0.253
**BMI**^**a**^		1.08	0.93,1.24	0.317
**Wound length**^**a**^		0.96	0.84,1.09	0.535

^a^Odds-ratio estimated by unit increase. A: standard dressing; B: Aquacel Ag Hydrofiber dressing; CT+RT: preoperative chemoradiation therapy.

### Microbiology

Culture of the surgical site in infected patients yielded growth of organisms (a total of 25 isolates), and similar proportions of infected patients in the two study groups (five of nine (55.5%) in the Aquacel Ag Hydrofiber group, and six of eleven (54.5%) in the control dressing group) had an identifiable microbiologic cause of infection. Gram-positive aerobic bacteria (15 isolates) outnumbered Gram-negative aerobic bacteria (10 isolates), and 20% of cultures were polymicrobial.

There were no significant differences in the frequency of isolating certain categories of organisms or particular organisms in the experimental group as compared with the control group.

### Adverse events

Investigators who were unaware of the patients’ group assignments assessed the seriousness of all adverse events; none was determined to be related to the study.

## Discussion

Randomized studies have compared the efficacy of different types [11-13] or doses [14,15] of systemic antibiotics for preventing SSIs; influence of different preoperative skin antisepsis agents was also investigated [5]. To our knowledge, this is the first randomized controlled trial addressing the efficacy of an ionic-silver-delivering antimicrobial dressing in lowering the SSI rate of patients having colorectal cancer elective open surgery.

Animal models have been used to study the performance and cytotoxicity of Aquacel Ag Hydrofiber dressing *in vivo*. In a full-thickness infected mouse wound model, Yates and colleagues showed that Aquacel Ag Hydrofiber lowered wound bacteria loads, obtained superior wound tensile strength, and accelerated epithelialization compared with untreated infected wounds [16]. Multiple clinical studies have been performed to assess Aquacel Ag Hydrofiber effectiveness for the treatment of a variety of acute and chronic wounds. Caruso *et al*. carried out a stratified, randomized prospective, though un-blinded study, comparing Aquacel Ag Hydrofiber and silver sulfadiazine in the management of partial thickness burns [17,18]. Aquacel Ag Hydrofiber was associated with significantly less pain and anxiety during dressing changes, significantly fewer procedural and opiate medications, significantly less burning and stinging during wear, and greater cost-effectiveness than silver sulfadiazine.

Other clinical studies on partial thickness burns treatment showed good wound healing, reduced pain, fewer dressing changes and better cost-effectiveness with Aquacel Ag Hydrofiber compared to standard treatment [19-21]. In patients with acute split-thickness skin-graft donor sites and acute traumatic wounds [22,23], Aquacel Ag Hydrofiber showed superior re-epithelialization rates and was associated with less pain on dressing removal in comparison with standard treatment. Jurczak and colleagues of the Aquacel Ag Surgical/Trauma Wound Study Group carried out a prospective, randomized clinical trial comparing pain, comfort, exudate management, wound healing and safety with Hydrofiber dressing with ionic silver (Aquacel Ag Hydrofiber dressing) and with povidone-iodine gauze for the treatment of open surgical and traumatic wounds [22]. At final evaluation, Aquacel Ag Hydrofiber dressing was significantly better than povidone-iodine gauze for overall ability to manage pain, overall comfort, wound trauma on dressing removal, exudate handling and ease of use. Rates of complete healing at study completion were 23% for Aquacel Ag Hydrofiber dressing and 9% for povidone-iodine gauze (*P*-value was non-significant).

There are several limitations of our study that deserve some mentions. First, results of this randomized trial are conflicting with those initially reported by our group in a Congress abstract [10], summarizing the data of a pilot study carried out in 100 patients consecutively receiving Aquacel Ag Hydrofiber dressing after elective colorectal cancer surgery. In that report, we observed an overall SSI rate of 4%, which is much less than the 15.5% we detected in this randomized trial. However, limitations of observational non-randomized studies are well known and emphasized, especially uncertainty about whether all confounders had been controlled for. Double-blinding was accurately provided in this randomized trial, and we can reasonably argue that initial results should be interpreted cautiously, particularly given the absence of blinding in that initial experience. Moreover, the usage of the secondary dressing to blind the study might have affected the overall performance of the antimicrobial dressing.

Second, the overall rates of SSI of 17.9% in this study are higher than those reported in some previous studies [24,25]; nevertheless, they are similar to the pre-study rates at the participating hospitals and those reported in other studies [26] and are lower than the rates reported in trials that used the CDC definition of infection and had adequate follow-up [2,12,13], as we did in this trial. In this study we universally enforced standard of care preventive measures (for example, administering systemic prophylactic antibiotics within the 30 minutes before the first incision was made and, if needed, clipping hair immediately before surgery) [27,28]. However, we did not use chlorhexidine-alcohol as the skin preparation agent; although the povidone-iodine we used possesses broad spectrum antimicrobial activity [27], a recent randomized controlled trial demonstrated that preoperative cleansing of the patient’s skin with chlorhexidine-alcohol is superior to cleansing with povidone-iodine for preventing SSI after clean-contaminated surgery [5]. This is probably related to its more rapid action, persistent activity despite exposure to bodily fluids, and residual effect [29].

Finally, the sample size of this trial could appear smaller than required, and the study globally underpowered to detect a significant difference in infections rates, even though the sample size was accurately determined on the basis of a pilot non-randomized prospective study. We were not able to enroll more patients in the timeframe we assigned to the study, as minimally invasive surgery (a factor excluding eligibility in this trial) has come to be more and more applied to surgical treatment of colorectal cancer. Superficial SSIs have been significantly reduced by the application of minimally invasive techniques [30]. Precise robotic movements and fine manipulation of tissues in a close and fixed operating field make especially rectal cancer surgery an important application of minimally invasive robotic surgery, and in fact robot-assisted low-anterior resection was reported by ours and other groups as a technically feasible and oncologically safe procedure for treatment of rectal cancer [31,32].

## Conclusion

In conclusion, Aquacel Ag Hydrofiber dressing showed a reduction in the overall SSI rate compared with a common postoperative dressing in elective colorectal open major procedures, although the registered infections rates were not statistically significant. Moreover, there were no significant differences between the two study groups in the incidence of superficial and deep infections of surgical wounds and, as expected, there was no difference in organ-space infection rate either. The finding that rectal cancer surgery is more prone to SSI compared with colon surgery confirms previous reports in the literature. No adverse events related to the study were detected. More trials are needed to definitely address this topic, testing new products developed specifically for surgical incisions, such as Aquacel Ag Surgical cover dressing (Hydrofiber Technology combined with skin-friendly hydrocolloid substrate), which provides a waterproof and viral and bacterial barrier that allows for flexibility and extensibility during body movement.

## Competing interests

This investigator-initiated trial was conceived by the authors, who also gathered the data, wrote the first and final versions of the manuscript, and decided to submit the paper for publication. All authors vouch for the completeness and accuracy of the data . Medical Researchers of ConvaTec (Princeton, NJ, US), manufacturer of the antimicrobial dressing studied, offered technical support, contributed to the design and conception of the study, and critically revised the manuscript but played no role in data collection or analysis. 'The authors declare that they have no competing interests.

## Authors’ contributions

All authors had full access to the data and substantially contributed to the analysis and interpretation of the data and the writing of the manuscript. All authors read and approved the final manuscript.
